# Substrate-specific binding of 8-oxoguanine DNA glycosylase 1 (OGG1) reprograms mucosal adaptations to chronic airway injury

**DOI:** 10.3389/fimmu.2023.1186369

**Published:** 2023-08-08

**Authors:** Lang Pan, Spiros Vlahopoulos, Lloyd Tanner, Jesper Bergwik, Attila Bacsi, Zsolt Radak, Arne Egesten, Xueqing Ba, Allan R. Brasier, Istvan Boldogh

**Affiliations:** ^1^ Department of Microbiology and Immunology, University of Texas Medical Branch, Galveston, TX, United States; ^2^ Horemeio Research Laboratory, First Department of Pediatrics, National and Kapodistrian University of Athens, Athens, Greece; ^3^ Respiratory Medicine, Allergology & Palliative Medicine, Department of Clinical Sciences Lund, Lund University and Skåne University Hospital, Lund, Sweden; ^4^ Department of Immunology, Faculty of Medicine, University of Debrecen, Hungary, Debrecen, Hungary; ^5^ Research Institute of Sport Science, University of Physical Education, Budapest, Hungary; ^6^ Key Laboratory of Molecular Epigenetics of Ministry of Education, School of Life Science, Northeast Normal University, Changchun, Jilin, China; ^7^ Department of Medicine, University of Wisconsin-Madison School of Medicine and Public Health (SMPH), Madison, WI, United States

**Keywords:** ROS, epigenetics, NFκB, SMADs, inflammation, remodeling

## Abstract

Recent advances have uncovered the non-random distribution of 7, 8-dihydro-8-oxoguanine (8-oxoGua) induced by reactive oxygen species, which is believed to have epigenetic effects. Its cognate repair protein, 8-oxoguanine DNA glycosylase 1 (OGG1), reads oxidative substrates and participates in transcriptional initiation. When redox signaling is activated in small airway epithelial cells, the DNA repair function of OGG1 is repurposed to transmit acute inflammatory signals accompanied by cell state transitions and modification of the extracellular matrix. Epithelial-mesenchymal and epithelial-immune interactions act cooperatively to establish a local niche that instructs the mucosal immune landscape. If the transitional cell state governed by OGG1 remains responsive to inflammatory mediators instead of differentiation, the collateral damage provides positive feedback to inflammation, ascribing inflammatory remodeling to one of the drivers in chronic pathologies. In this review, we discuss the substrate-specific read through OGG1 has evolved in regulating the innate immune response, controlling adaptations of the airway to environmental and inflammatory injury, with a focus on the reader function of OGG1 in initiation and progression of epithelial to mesenchymal transitions in chronic pulmonary disease.

## 1 Introduction

Airway remodeling may develop during recurrent lung damage, arising due to chronic inflammatory processes and aberrant tissue restoration. The reoccurring injury and repair produce structural changes in tissue leading to non-physiological airway configuration, airway narrowing and ultimately permanent airflow impediment. Loss of airway function has been characterized by an excessive deposition of extracellular matrix (ECM) and tissue scar formation. Cell types primarily responsible for ECM accumulation are the myofibroblasts (1). After injury-induced wound healing, myofibroblasts are usually eliminated by apoptosis as epithelialization is completed. However, in the pathological setting of repeated injury, myofibroblasts persist and continue to produce collagen, which exacerbates fibrosis. In addition, due to oxidative stress evoked during the generation of growth factors and cytokines, epithelial cells may transition or differentiate into myofibroblasts through multiple biochemical changes, a process known as type II epithelial to mesenchymal transition (EMT) (2). During the EMT process, epithelial cells undergo transcriptional reprogramming, changes in polarity and loss of cell-to-cell contacts. These cells show altered responsiveness and expression of myofibroblast markers, such as alpha smooth muscle actin (αSMA) and fibroblast-specific protein 1, instead of epithelial-specific markers that affect innate immune signaling (1, 3). This review explores how airway epithelial cells dynamically respond to immune signaling through the DNA repair protein 8-oxoguanine DNA glycosylase 1 (OGG1), which recognizes oxidatively modified guanine bases, and OGG1’s involvement in changes to the transcriptional profiles of epithelium to reprogram tissue homeostasis.

## 2 An overview of the repair attributes of OGG1

Each of the nucleobases reacts differently with electrophiles, with guanines (Gua) considered the most easily oxidized nucleobase. Oxidation of Gua in DNA at C8 produces 7,8-dihydro-8-oxoguanine (hereafter called 8-oxoGua), whereas both 5’ Gua and 3’ Gua are more reactive (4, 5). 8-oxoGua is one of the most abundant purine-derived DNA lesions and has received appreciable attention due to its potential role in mutagenicity. Because the *syn* conformation is thermodynamically preferred for 8-oxoGua, it is able to form a stable Hoogsteen base-pair with adenine rather than cytosine, leading to transversion mutations after replication (6). OGG1 displays a marked preference for the 8-oxoGua:cytosine base pair as a substrate, albeit tolerating substitutions opposite the damaged base (7). OGG1 also recognizes 8-oxoadenine paired with cytosine or 5-methylcytosine, yet this recognition depends on the opposite base (8).

Although 8-oxoGua differs from normal guanine by only two atoms, OGG1 can recognize and remove it very efficiently. The specificity of OGG1 not only depends on recognizing 8-oxoGua itself, but also complementary bases, which are checked before the catalytic reaction. A major question for base excision repair (BER) glycosylases is how they locate damaged bases in nucleosomes. It is presently believed that OGG1 does not require cofactors or energy for such activity, because it scans DNA by thermal diffusion (9). Moreover, oxidative stress re-locates OGG1 and other BER proteins to open chromatin within regions rich in transcription factors (10). This raises the possibility that OGG1 may act in concert with chromatin remodelers for preferential recognition of its substrate in transcriptionally active DNA (11). Several cofactors can promote efficient recruitment of OGG1 to chromatin (12), including histone marks, protein cofactors or even 8-oxoGua itself. Many aspects of repair processes are covered by comprehensive reviews published by Dr. Dizdaroglu (13), Dr. Mitra (14), Dr. Radak (15) and Dr. Lloyd (16).

## 3 OGG1 as an epigenetic reader and eraser

**Figure 1:**
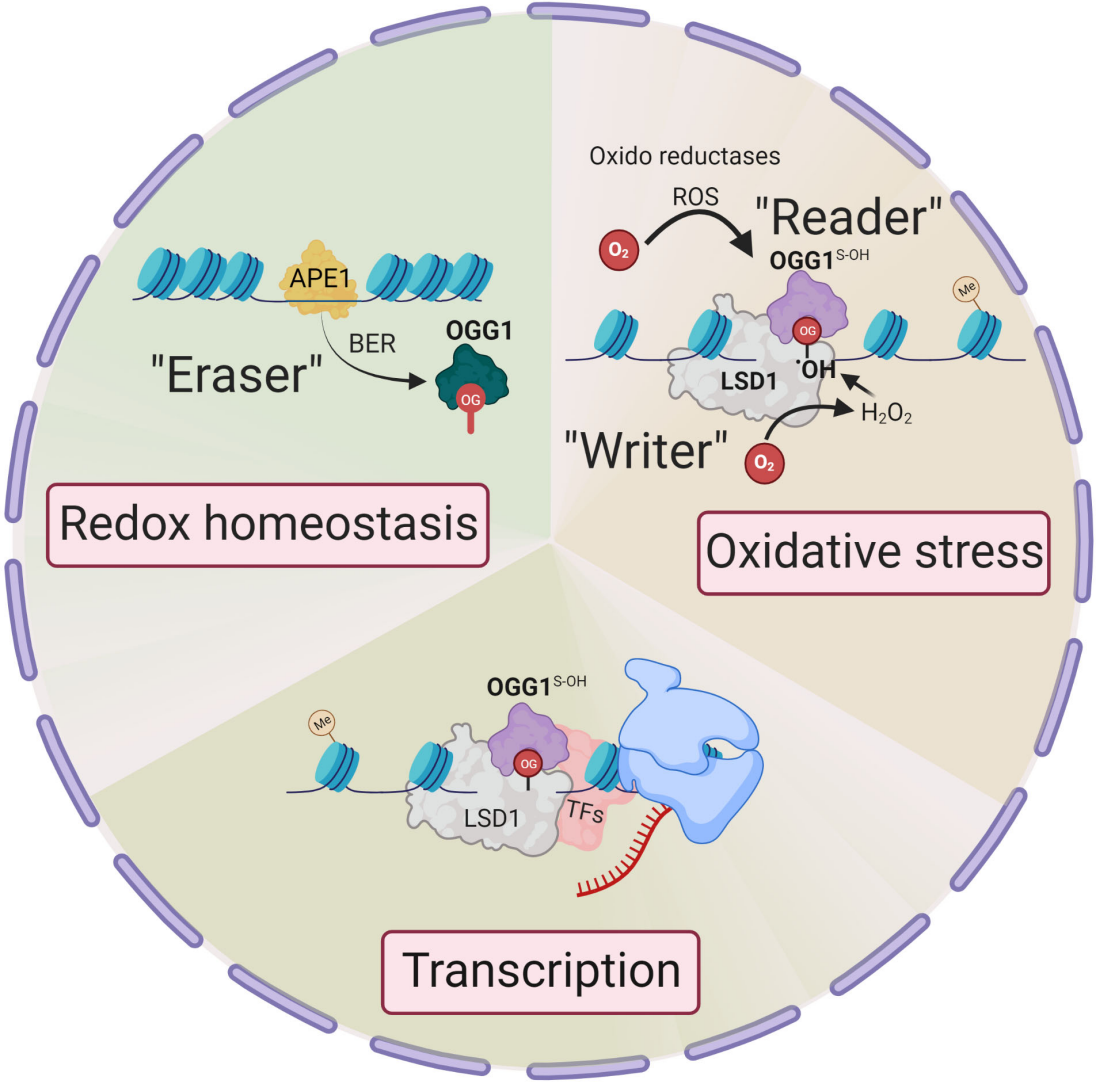
Epigenetic changes in OGG1 dependent gene expression. Signaling through ligand-receptor interaction activates local histone demethylation by LSD1. Upon interaction of LSD1 with mono-methylated or demethylated lysine, the flavin-dependent monoamine oxidase activity, generate an imine intermediate and FADH_2_, which is then oxidized to FAD by O_2_, resulting H_2_O_2_. H_2_O_2_ → •OH oxidizes Gua to 8-oxoGua. Thus, LSD1 selectively generates (writes) 8-oxoGua at the site where transcriptional initiation complex is assembled. ROS by various oxidoreductases also generate intrahelical 8-oxoGua especially in G:C-rich promoters. 8-oxoGua is “read” by oxidatively modified OGG1 (OGG1^S-OH^) (“reader”) and facilitates transcription factors DNA occupancy and gene expression. Upon cellular redox homeostasis re-established, reduced OGG1 removes 8-oxoGua (“eraser”) and DNA is repaired through BER pathway to maintain genome integrity.

Unlike enzymatic DNA methylation, it is challenging to envision selective Gua oxidation by reactive oxygen species (ROS). Lysine-specific demethylase 1 (LSD1) is a nuclear flavoenzyme that generates ROS during demethylation of H3 lysine (17). The studies from LSD1provide an integrated mechanism whereby 8-oxoGua is generated at both enhancer and promoter sites which, in turn, recruit OGG1 and DNA repair components, thereby activating estrogen-induced gene expression (18, 19). This is notable, as histone H3 Lys4 trimethylation sites are proposed to be oxidant-sensitive epigenetic marks, with their global reduction upon cell stress augmenting gene expression (20). Therefore, the genomic response to oxidation is spatially and temporally governed, with OGG1 locating chromatinized substrates involved in the histone code. In this regard, LSD1 can be deemed an epigenetic “writer” that writes 8-oxoGua locally at active transcription sites (21) (**Figure 1**).

Pioneering observations of oxidative base modifications functionally generated in gene promoters have been investigated by the Gillespie Laboratory (22). They corroborated that targeted oxidative lesion formation within hypoxia-inducible factor-1 (HIF-1) response elements of the vascular endothelial cell growth factor (VEGF) gene promoter could contribute to hypoxic signaling. These data were followed by numerous studies, including studies from the Burrows Laboratory (23) that defined 8-oxoGua as an epigenetic mark. Our views of 8-oxoGua as an epigenetic mark and of OGG1 processing *trans*-regulatory function were catalyzed by the advancement in technologies that mapped 8-oxoGua in the genome. A series of studies associated 8-oxoGua with regulation of transcription, especially from enhancers and promoters (24–27). Pezone et al., also highlight oxidative modification to 8-oxoGua in DNA as an active response to initiate the EMT transcriptional program (28), further supporting the epigenetic role of 8-oxoGua in gene expression processes. OGG1 reading 8-oxoGua locally can function as a rheostat for functional output from that restricted locus. Given that response to oxidative DNA damage is pleiomorphic and probably extends to other transcription factors, these events link the histone code to a broadly used redox strategy that circumvents 8-oxoGua acting epigenetically through controlled OGG1 function. To this end, OGG1 can be considered an epigenetic “reader” and an “eraser”. Recent studies demonstrate that OGG1 is important in oxidative stress-generated DNA demethylation (29). OGG1 stimulates DNA demethylation by cooperating and engaging with ten-eleven translocation 1 protein (TET1) at the site of an 8-oxoGua lesion. OGG1 knockdown makes cells tolerant to ROS-induced DNA demethylation, while transgenic over-expression of OGG1 makes cells vulnerable to DNA demethylation by ROS. These data not only illustrate the importance of BER in DNA demethylation but also reveal how the DNA demethylation signal is transferred to downstream DNA demethylation enzymes.

### 3.1 Reversible cysteines oxidation switches OGG1 eraser function to reader

The repair-coupled function of OGG1 is examined by a ROS-induced recruitment from a soluble nucleoplasmic localization to the nuclear matrix, where OGG1 colocalizes with nuclear speckles (10). Its relocation is specific for oxidative stress as it is prevented by the presence of antioxidant compounds (30). In addition to protein localization, enzymatic activity also can be regulated in response to changing redox conditions. Reversible modification in cysteines highlights their ability to function as redox switches that alter protein function (31). The reaction of ROS with OGG1 cysteine residues transiently suspends its enzymatic activity while preserving recognition and initial steps in substrate processing (32–35). Upon re-establishing a cellular redox state, enzymatic activity of OGG1 is completely recovered (36). Germane to this are data demonstrating that TNFα exposure induced transient oxidation at cysteine residues of OGG1 with concomitant inactivation of 8-oxoGua excision (37, 38). Another report demonstrates that C28 oxidation-induced OGG1 dimerization, which does not impede substrate recognition but suppresses OGG1 catalytic activity at 8-oxoGua, results in enhancement of Myc recruitment to its E-box promoter recognition sequences (39). These data imply that under oxidizing or inflammatory conditions, OGG1 is responsible for increased expression of Myc target genes. These studies provide intriguing evidence that redox chemistry of cysteines may represent a paradigm by which OGG1 transiently functions as an epigenetic reader, and upon restoration of the intracellular redox environment acts as an eraser, which guarantees the integrity and stability of the genome. Another possibility is that the 8-oxoGua erasing function of OGG1 is coupled with overlapping DNA repair and gene regulatory events. In this model, the epigenetic mark 8-oxoGua in promoters and enhancers is erased by OGG1, followed by the repair intermediates, such as AP-sites binding of AP endonuclease1, facilitating assembly of transcriptional machinery (23, 40). This model also permits cells to achieve two vital tasks, accommodating oxidatively modified DNA bases for timely transcriptional response.

### 3.2 Locating epigenetic marks and structural DNA rearrangement induced by OGG1

OGG1 function is facilitated by diffusion, a mechanism that primarily involves protein sliding (41, 42). OGG1 scans DNA by thermal diffusion, moving along DNA by sliding that allows exploration of modified bases around points of successive DNA encounters (43). On the basis of structural studies, OGG1-8-oxoGua associations involve at least two steps, typically an initial enzyme–substrate interaction followed by DNA structural remodeling (44). OGG1 induces marked rearrangement of the DNA structure around the epigenetic mark, eviction of which leaves the complementary cytosine estranged in the helix, and a sharp kink (~70°). This subtle but significant DNA topography change helps us understand how OGG1 acts upon 8-oxoGua in the context of chromatin, where local DNA flexibility supports OGG1’s association with other proteins, including transcription factors. OGG1 is programmed via its structure to recognize and act upon a particular lesion, and this conformational behavior has furthermore suggested ways to modulate OGG1 function in terms of substrate specificity, repair fidelity, handoffs, and the coordination of different repair steps, as previously reviewed (45, 46).

### 3.3 OGG1 facilitates DNA occupancy of the transcriptional machinery

Because 8-oxoGua is virtually identical to the native Gua base as shown by X-ray crystal and NMR structural studies (47, 48), it is expected that 8-oxoGua alone does not affect the DNA-binding efficiency of transcription factors (TFs). However, it appears that the binding affinities of TFs (like NFκB1p50, AP1, SP1 and CREB) are affected by 8-oxoGua itself (49–53). It is thus not surprising that considerable efforts were made to better understand the role of OGG1 at 8-oxoGua sites in a physiological context. The ability of TFs to bind to their consensus sequence is impaired by oxidative events (54, 55). Our studies filled this gap by providing a mechanism for coordination of intrinsic DNA binding proteins, like OGG1, to act as pioneer factors for TF binding under oxidative stress (38). When compared to a wild-type consensus oligonucleotide, levels of p50-p50 and p50-RelA(p65) bound to 8-oxoGua containing DNA are higher by 15 to 25-fold in the presence of OGG1, respectively. Moreover, OGG1 significantly shortens the time required for the DNA occupancy of homo- and heterodimeric NFκB. OGG1 recognizes oxidized substrates clustered around the NFκB binding element, which could accommodate local DNA topography suitable for binding, thus increasing NFκB binding efficiency and activating transcription. In the chromatin context, genome-wide binding of OGG1 is also mapped to illustrate stimulus-driven association of OGG1 with NFκB (56). Another study using chromatin immunoprecipitation (ChIP) assay shows that OGG1 reading 8-oxoGua accommodates phosphorylated SMADs complex binding to SMAD binding elements of pro-fibrotic genes, such α-SMA, fibronectin (FN) and collagen (COL), thereby facilitating gene activation (57). The physiological significance is striking, as OGG1 reading 8-oxoGua is temporally utilized to skew the repair function to trigger transcription, thereby repurposing OGG1 for the immune response.

## 4 Role of OGG1 in mucosal immune landscape

An emerging topic in the field of mucosal immunology is the influence of epithelial cells on subsequent adaptive responses (58–60). Functionally, epithelial cells are equipped with a variety of receptors including pattern recognition receptors, which are necessary and sufficient to trigger the innate immune response (IIR) (61). Once activated, epithelial cells can produce cytokines and chemokines, which are responsible for autocrine and paracrine regulation of homeostasis. Therefore, an obvious hierarchy exists in the capacity of the epithelium to set the threshold for restoring lung homeostasis.

Small airway disease is a common feature of pulmonary pathology. With the advances of single cell sequencing, heterogeneous epithelial and fibroblast subpopulations in fibrotic lungs were discovered (62). The various epithelial and mesenchymal lineages indicate that each lineage has a distinct spatial address and transcriptional profile leading to unique niche regulatory functions (63), with the plasticity of the distal lung epithelium driving pathologic epithelial remodeling and ECM expansion occurring at the peripheral regions and slowly progressing inward. Genetic lineage tracing studies in murine models of airway fibrosis have shown that peripheral fibrotic cells originate from alveolar epithelial cells during abnormal tissue repair (64). It is becoming increasingly apparent that distal lung epithelium, or small airway epithelium, could become a potential therapeutic target in inflammatory lung disease.

### 4.1 OGG1 facilitates the expression of inflammatory mediators

Comprehensive ChIP-coupled next generation sequencing analyzed OGG1 enrichment after airway lung epithelial cells were exposed to a single dose of TNF-α (56). In controls, NFκB/RelA DNA occupancy was examined, and its enrichment peaks were mapped to the human genome and compared to OGG1. The OGG1 enrichment peaks (over ten thousand) were primarily localized to promoter regions adjacent to transcription start sites (TSS), and far fewer enrichment peaks were localized to exons, introns, untranslated regions (UTRs), or intragenic regions. Similar distribution of NFκB/RelA was observed, which was enriched in over 8000 *cis*-acting target sequences. Importantly, the allocations of the OGG1 and NFκB enrichment peaks relative to TSS were similar. Systems biology-level approaches through hierarchical structure and relationship analysis shows that the primary enriched gene ontology (GO) term was inflammation and immune response (IIR) processes. The most significantly modulated processes were the positive regulation of cytokine production, including TNFα, IL6, IFNs, C-X-C motif chemokine ligands and IL1, the heterodimeric IL23 and IL17. In independent studies (65), RNA-seq data clearly showed transient expression of C-C, C-X-C motif chemokines, cytokines and interleukins, which facilitates OGG1-driven recruitment of site-specific transcription factors (NFκB, AP1, SP1) to promoters.

Adaptations in OGG1 substrate-specific reading support the capacity of TFs binding, resulting in an epigenetic switch that initiates a chain of transcriptional events to induce IIR. Following this logic, it is reasonable to justify the attenuated immune response observed in *Ogg1* knock out (KO) mice in inflammatory models. Compared with wild-type counterparts, *Ogg1* KO mice are resistant to LPS-induced tissue damage and organ dysfunction, with decreased cytokine and chemokine levels in body fluids (66). In the model of allergic inflammation induced by ovalbumin (67), *Ogg1* KO mice also exhibit lower IIR and infiltration of allergic inflammatory cells in the airway, manifested as lower expression of pro-inflammatory mediators and tissue damage. Functional inactivation of OGG1 in sensitized and ovalbumin-challenged mice resulted in decreased expression of proinflammatory cytokines and chemokines, goblet cell hyperplasia and mucus production, with significantly lower recruitment of eosinophils and other immune cells to the lungs (68). The essential role of OGG1 in ROS-induced proinflammatory gene expression has been demonstrated by siRNA-mediated silencing of OGG1 in airway epithelium (69), small-molecule inhibition of OGG1 substrate binding (70–72), and functional ablation of excision activity (73). Absence of OGG1 expression or its pharmacological inhibition by TH5487 or SU0268 leads to decreased inflammatory gene expression, thus blocking airway hyperresponsiveness in allergic reactions and acute inflammation (68). Furthermore, robust OGG1-dependent influx of neutrophils was observed from 6 hours post-challenge, with a peak at 24 hours, and by 72 hours the number of neutrophils returned to pre-challenge levels (37, 69, 74). Together, these studies establish the frameworks within which OGG1 contributes to IIR and to adaptive responses by regulating the expression of inflammatory mediators at the transcriptional level.

## 5 OGG1 contributes to inflammatory tissue remodeling

Epithelial-immune interactions raise a response to promote repair of damaged structures, a process that often provides existing tissue structures with modifications that optimize defense from structural perturbations (75, 76). This is the case with the generation of ECM proteins that are deposited around the damaged area, which is achieved via consecutive and overlapping phases of epithelial-mesenchymal interactions, cell proliferation and tissue remodeling (77). Although inflammatory tissue remodeling is essential for host defense and allows for efficient healing and reestablishment of homeostasis, unabated inflammation or otherwise abnormal remodeling is one of the drivers of fibrosis. However, the mechanism(s) that underlie sensing and reacting to these changes that are ultimately required to limit the repair process and to resolve inflammation, is not well understood.

**Figure 2:**
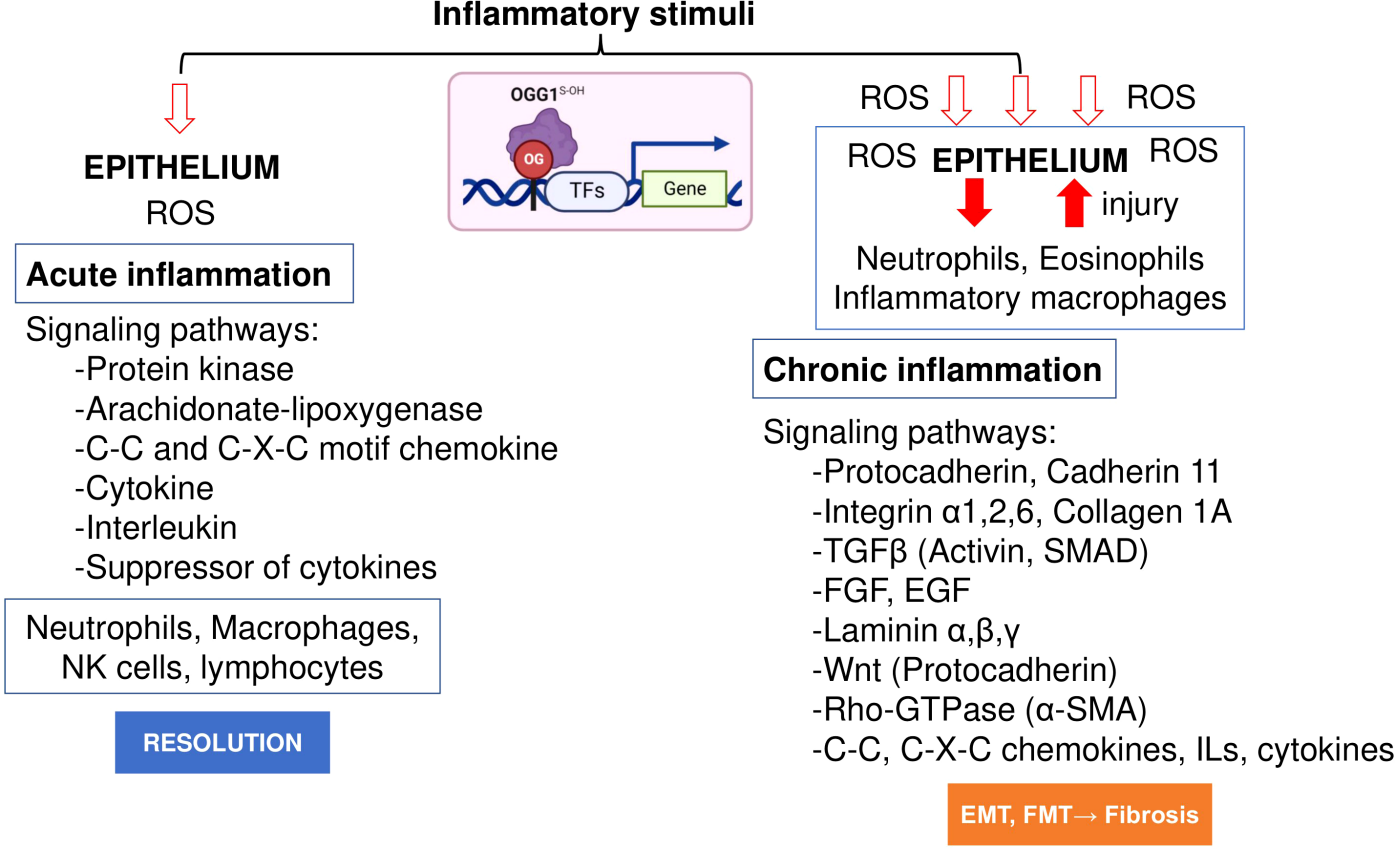
OGG1 dependent signaling pathways in acute and chronic mucosal inflammation. OGG1 reads 8-oxoGua in epithelium, first responding to insults and continuing throughout the repair process. OGG1 facilitates the DNA occupancy of transcription factors (e.g., NFκB), thereby subsequent expression of pro-inflammatory mediators in acute inflammation. In repeated injury, OGG1 reprograms immune landscape toward pro-fibrotic and ECM gene expression that induce damage-responsive epithelium transition by facilitating DNA occupancy of SMADs. In these studies, experimental animals were challenged once or repeatedly with pro-inflammatory agent. RNA extracted from lungs were RNA sequenced and analyzed at system levels. Animal studies were performed according to the NIH Guide for Care and Use of Experimental Animals and approved by the University of Texas Medical Branch Animal Care and Use Committee (approval no. 0807044D). OG, 8-oxoGua; TF, transcription factor, FGF, fibroblast growth factor; EGF, epidermal growth factor; Rho-GTPases, Ras Homolog family member; SMAD, Mothers against decapentaplegic homolog.

Initial work by Luo et al. (78) and Aguilera et al. (65) examined the role of OGG1 in inflammatory airway remodeling in a mouse model. To gain insight into gene expression, whole transcriptome analysis was performed. Studies were supported by biomolecular and tissue histological characterization, including epithelial alterations, smooth muscle mass, and ECM/collagen deposition in the airways. Compared to acute inflammation (single challenge), gene expression induced after multiple inflammatory challenges as a model for chronic inflammation highlighted quantitatively and qualitatively different expression profiles (**Figure 2**). The highly significant biological processes dependent on OGG1 expression in chronic inflammation were developmental processes, system development, cellular processes, cell adhesion, biological adhesion, cell communications and cell-to-cell adhesion. However, chronic inflammation (in contrast to acute) has been correlated to histological changes represented by extensive epithelial metaplasia, formation of trophic units, collagen deposition and smooth muscle hyperplasia primarily associated with smaller airways. Notably, the significance of immune system processes was the lowest (*P* = 3.69 × 10^10). The significantly overrepresented protein classes included ECM proteins, cell adhesion molecules, structural proteins, actin family cytoskeletal proteins, ECM structural proteins, cadherins, protease inhibitors, metalloproteinases, serine protease inhibitors, cell junction proteins, cell adhesion, in line with tissue histology, cell morphology and remodeling. Although these experiments utilized the rodent model, hierarchical clustering using gene-by-environment (GEN-E) showed that protein expression profiles and pathways are similar to those involved remodeling in humans (like the highly significantly expressed protocadherin, cadherin, catenin, integrin, laminin, α-actin, Rho GTPase, transforming growth factor-beta (TGFβ), epidermal growth factor (EGF), the TGFβ superfamily protein, the growth differentiation factor 2, myosin heavy polypeptide 1 to 13 and Wingless-type MMTV integration site family (Wnt), collagen type I to XVII) (**Figure 2**).

OGG1-dependent inflammatory responses and consequent tissue damage involve not only ROS generation, but also increased secretion of tissue remodeling relevant cytokines, chemokines and growth factors, among them, TGFβ (79). TGFβ is renowned as a pleiotropic factor that both promotes and inhibits processes like inflammation and proliferation, depending on the physiological context. In the context of fibroblasts, TGFβ activates NOX-4 and generates H_2_O_2_ that is required for myofibroblast differentiation, ECM production and contractility (80). In the immune system, TGFβ mediates naïve T cell transition toward immunosuppressive phenotypes, decreased inflammation upon repeated challenges (81). Excessive release of TGFβ can be activated by mechanical tension-induced cell contraction (82), which occurs during physical stretch or constriction events that result from gasping or when using mechanical ventilation in patients with acute respiratory distress syndrome (ARDS). Mechanical tension induces tissue injury, causing disruption of the cell-cell junctions or ECM architecture. This also imposes supraphysiological mechanical forces on the epithelial cells. Interestingly, mechanical stress resultant from inflammatory exudates in lower airways generates oxidative DNA damage, as detected by the presence of 8-oxoGua (57, 83), suggesting that 8-oxoGua is actively generated by TGFβ signaling. For epithelial repopulation, integrin αvβ8-mediated activation of TGFβ inhibits airway epithelial cell proliferation (84). The integrin αvβ8 is expressed in basal cells, which are the major contributor to epithelial regeneration by giving rise to differentiated airway epithelial cell types (85). Therefore, the presence of TGFβ represents a scenario restricted re-epithelization from dedicated stem cell lineages (86), indicating that a different sub-population performs a significant level of repair, which is essential for either response to acute injury or for retaining architectural integrity.

### 5.1 Requirement for OGG1 in TGFβ-induced mesenchymal phenotype

**Figure 3:**
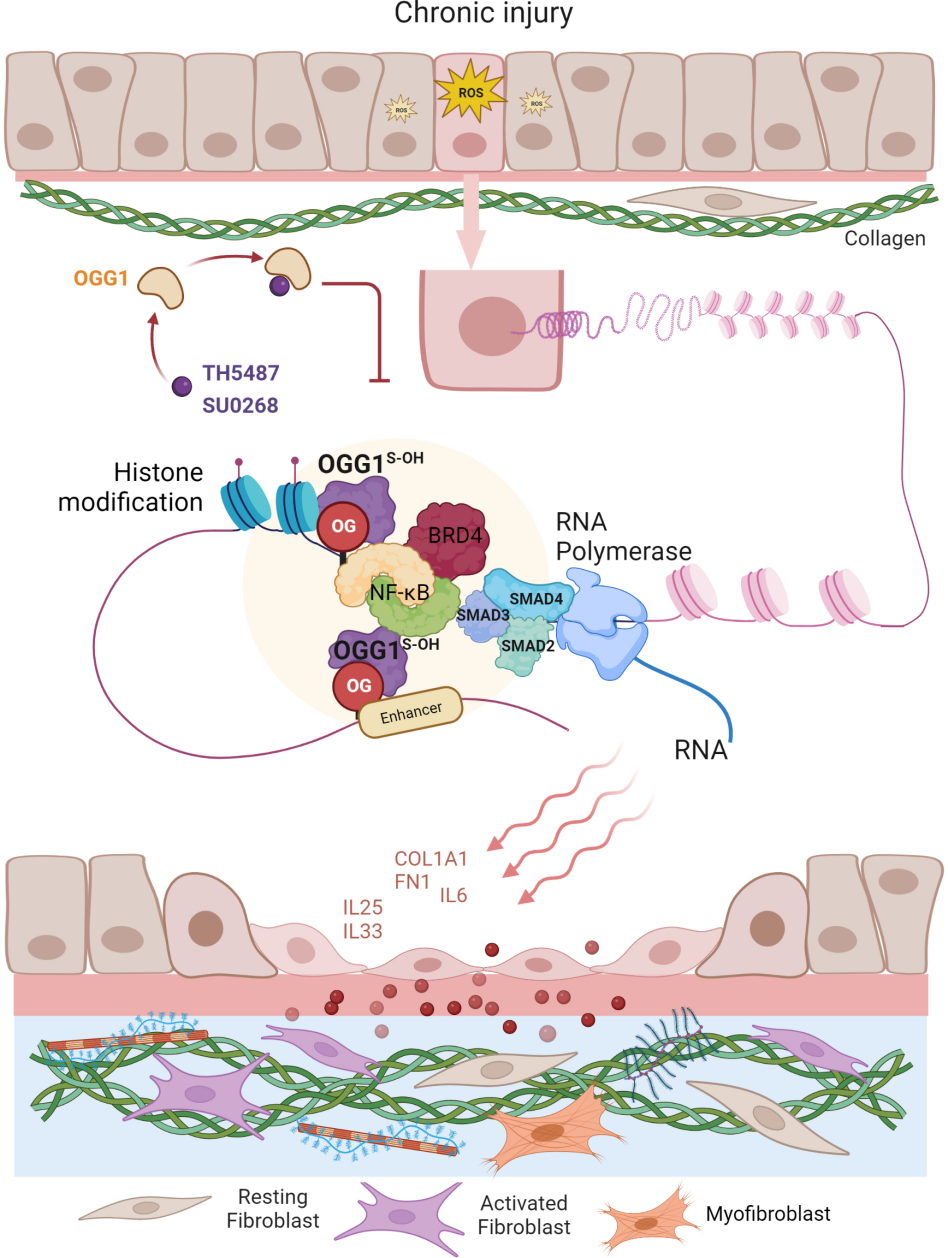
Graphical depiction of OGG1 function in the development of airway remodeling. In response to repeated exposures to environmental pollutants, irradiation, aeroallergens or respiratory virus infection, epithelial cells undergo EMT and the subepithelial fibroblasts are one of several mesenchymal lineages that transition into myofibroblasts (FMT) expressing αSMA and COL1A1. Both the EMT and FMT paralleled with IIR and initiated by growth factors (e.g., TGFβ, EGF). IIR and both EMT/FMT executed by molecular events surrounding OGG1-facilitated DNA occupancy of transcription factors (e.g., SMADs, NFκB) and chromatin remodelers (e.g., BRD4, LSD1). Changes in cell stiffness and matrix metalloproteinase secretion, all initiated from EMC produced by epithelial injury/repair. Epithelial barrier disruption induces secretion of growth factors and fibrogenic cytokines (periostin, IL-17, IL-11). TGFβ activates signaling cascades that result in fibroblast motility, anti-apoptosis, and expression of ECM proteins, FN1 and COL1.

The ability of epithelial cells to transition into mesenchymal cells, either partially or fully, illustrates the inherent plasticity of the epithelial phenotype (87). In a recent study by Pan et al. (57), small airway epithelial cells in culture could phenotypically convert to spindle-shaped mesenchymal cells, by a mechanism that involved OGG1-mediated molecular reprogramming in response to TGFβ exposure (**Figure 3**). Cellular plasticity in EMT requires transcriptional networks mediated by key transcription factors, of particular importance is SMAD3 in the SMAD complex. Like most TFs that are quiescent in steady state, SMAD3 is activated by phosphorylation to translocate into the nucleus where it binds to *cis*-regulatory regions of target genes in the chromatin. The consensus motif recognized by SMAD3 was originally identified as an 8 bp palindromic DNA sequence (5′-GTCTAGAC-3′) (88), where the 4 bp half site (5’-GTCT-3’) represents the minimal binding sequence for SMAD3 (89). The 5’-GC SMAD binding elements GGC(GC)|(CG) were shown to be more prevalent in SMAD3 target sites (90), yet alone not sufficient to confer recognition specificity. Moreover, the chromatin architecture restricts SMAD3 DNA occupancy. TGFβ treatment alters histone modifications at enhancers eliciting chromatin opening and enhancer activation (91), which are required for SMAD sequence occupancy. Critically, high-affinity and high-specificity recruitment of SMADs to DNA usually requires additional pioneer factors, which alter DNA structure to accommodate transcriptional initiation (92). We and others have shown that DNA oxidation marks an upstream event that triggers SMAD3 targeted genes (28, 57). TGFβ treatment activates LSD1 generating ROS locally, writing 8-oxoGua into the *cis*-regulatory regions in proximity to SMADs binding sites. OGG1 reads 8-oxoGua substrates without delay, making DNA conformational changes and promoting SMAD complex assembly into the transcription machinery.

To address the role of OGG1 in fibrotic processes in lungs, mice were challenged with TGFβ intranasally and TH5487 was added intraperitoneally to pharmacologically inactivate OGG1 (57). TGFβ generated high levels of 8-oxoGua in the genome, which correlated well with OGG1 enrichment on promoters of fibrotic genes. Studies have identified expression of EMT/FMT genes that were dependent on functional OGG1. These include TGFβ/SMAD3-targeted genes *Col1a2*, *Fn1* and *Vim*, Keratin 14 (Krt14, which is absent in distal airways of healthy lungs, but increased specifically in distal airways and alveolar regions of idiopathic pulmonary fibrosis (IPF) lungs) (93), Nodal growth differentiation factor (TGFβ super family protein, activator of SMAD family transcription factors), SRY-box containing gene 10 (Sox10, transcription factors involved in development and in cell fate outcomes). In line with gene expression data, immunoblotting shows that inactivation OGG1 by TH5487 decreased levels of proteins involved in the mesenchymal phenotype and pronounced epithelial characteristics. ChIP analysis showed that these genes were regulated by OGG1-dependent recruitment of SMAD3, Etv4 and NFκB to promoters. GO cluster analysis revealed that the regulated pathways are significantly involved in EMT and ECM organization networks. Lung histology clearly showed OGG1-dependent collagen deposition, which was decreased by TH5487. Moreover, TGFβ signaling induced C-terminal phosphorylation of SMAD2 and SMAD3 were not significantly altered with TH5487 treatment. These data together suggest that OGG1 reading genomic substrates triggers distinct cell-behaviors in the lung. When redox signaling is activated in small airway epithelial cells, the repair function of OGG1 is repurposed to transmit acute inflammatory signals accompanied by cell state transitions, modifying ECM. Epithelial-mesenchymal interactions and epithelial-immune effects act in concert to establish a local niche that instructs the mucosal immune landscape (**Figure 3**). If the transitioned cell state governed by OGG1 remains responsive to chemo-attractants instead of commencing differentiation, the collateral damage provides positive feedback to inflammation, and thus supports a role for inflammatory remodeling as one of the drivers in chronic inflammatory pathologies (60). OGG1-driven adaptive gene expression through epigenetic programming shapes the microenvironment of small airways under oxidative stress conditions.

### 5.2 Increased OGG1 expression during fibrotic processes

In TGFβ- or bleomycin-induced fibrosis, significant increases in OGG1 protein levels were documented in lung lysates (57, 94). In the same study, supraphysiological OGG1 levels were recorded after repeated TGFβ treatment using primary human lung fibroblast and murine-derived fibroblast cells, which paralleled with increased cell migration and with an enhanced wound healing capacity of the cells. Importantly, in lung sections of IPF patients, OGG1 immunoreactivity is significantly higher than in healthy controls, supporting the functional roles of OGG1 in fibrotic disease (94). Moreover, adeno-associated virus-driven OGG1 overexpression in rodent lungs promoted EMT in alveolar epithelia and resulted in progressive fibrosis (95). Notably, TH5487 significantly decreases OGG1 levels, which may be due to NEDD4 Like E3 Ubiquitin Protein Ligase (NEDD4L)-mediated OGG1 degradation. However, levels of DNA glycosylases (Neil2, Neil1 and Mth1) showed no change (57), highlighting OGG1 as a specific target. Increased OGG1 expression was observed in bleomycin-exposed mice and TGFβ exposed cells in culture (96). The increased expression of OGG1 promotes cell migration, while OGG1 depletion decreases the migratory ability. Expression of the transformation-associated markers, vimentin and αSMA, were also increased by OGG1. Taken together, these observations raise the possibility that the increase in OGG1 levels is not secondary, but rather tightly related to lung injury, tissue repair, and fibrotic processes. Although further studies are required, we can conclude several points. First, OGG1 is necessary for reading the epigenetic mark 8-oxoGua to modulate gene expression, the outcome of which has essential roles in tissue repair related to EMT. Second, OGG1 erasing oxidatively modified lesions ensures that epigenetic regulation, a highly dynamic process, facilitates rapid and transient phenotypic changes.

An important question is how OGG1 expression is regulated upon TGFβ exposure, particularly under pro-fibrotic/fibrotic conditions. Analysis of the *OGG1* promoter revealed the lack of TATA or CAAT boxes, implying a promoter type similar to constitutively expressed housekeeping genes. However, signal transduction, such as ROS signaling, can modulate the activity of promoters of housekeeping genes (97). Both bleomycin and TGFβ are strong inducers of oxidative stress, in fact, a large amount of evidence hints that ROS controls TGFβ and bleomycin signaling through various TFs, including SMADs (98–100). Importantly, the *OGG1* promoter region contains an antioxidant response element with a binding site for NF-E2-related factor 2 (Nrf2), suggesting that redox signaling upregulates OGG1 expression.

### 5.3 Potential clinical utility of OGG1 inhibitors in treatment of fibrosis

Chronic injury to the airway epithelium, combined with altered restoration capacity and fibroproliferative responses leads to IPF, an incurable condition characterized by increased lung stiffening and scarring, with an average survival rate of three years post-diagnosis (101). IPF is defined by fibroblast and myofibroblast overactivation resulting in excessive ECM deposition around alveolar walls, leading to significant reduction in alveolar spaces (102). In the absence of OGG1 function, the production of ECM proteins (like FN1, VIM and COL1A1) is inhibited within these transitioned cell states. *Ogg1* KO or inhibition of its reader function with TH5487 can be utilized in ameliorating oxidation-induced inflammation (71, 103), and to treat pulmonary fibrosis (94, 95) or allergic asthma (68). In bleomycin-induced lung injury, OGG1 KO mice show decreased collagen deposition and tissue damage (96). *OGG1*-targeting siRNA and inhibition (TH5487) of OGG1 significantly inhibit pro-migratory effects of TGFβ. Bioanalytical liquid chromatography tandem mass spectrometry data-independent acquisition (LC-MS/MS DIA) analysis showed significant down-regulation of OGG1-dependent GO terms, including collagen biosynthesis processes, wound healing involved in inflammatory response, endothelial cell proliferation, collagen metabolic processes, response to fibroblast growth factor, regulation of cytokine production, wound healing, and response to wounding in fibrotic tissue (94). These studies suggest that TH5487 is clinically relevant to the observed decreases in fibrotic processes. The convergence of signaling pathways from chromatin modifications and intrinsic DNA binding proteins is essential for gene expression related to inflammatory tissue remodeling. The regulation of NFκB typically controls proinflammatory gene expression, both alone and in concert with the SMADs cascade in response to injury, and their potential for pathway convergence explains how inflammatory tissue remodeling can be manipulated at the transcriptional level and targeted by OGG1.

Currently, there are two FDA-approved therapeutics available for the treatment of IPF. Nintedanib (Ofev^®^) targets tyrosine kinases by binding to the ATP-binding pocket, thereby blocking signaling cascades that result in the proliferation and migration of lung fibroblasts (104). The second therapeutic pirfenidone (Esbriet^®^) decreases TGFβ expression and inactivates downstream signaling, including phosphorylation of SMAD3 (104–106). Both nintedanib and pirfenidone have documented limitations, such as modest slowing of disease progression that is often coupled with poor tolerability (106–108), which highlights the need for novel therapeutic strategies. The effects of both drugs were compared to TH5487 in a bleomycin-induced mouse model of IPF (94). Similar to nintedanib, TH5487 decreased TGFβ-induced wound healing, fibroblast migration, morphological changes in epithelial cells and F-actin reorganization, as well as production of collagen around small airways (57, 94). Interestingly, TH5487, but not nintedanib or pirfenidone, significantly decreased TGFβ levels in bronchoalveolar lavage fluid (BALF), plasma, and lung homogenates. Treatment with TH5487 also decreased collagen deposition and structural deformation of the alveoli, indicating that TH5487 may be an effective inhibitor of bleomycin-induced fibrosis as characterized by histological lung damage. Finally, TH5487 decreased immune cell recruitment, levels of proinflammatory and profibrotic mediators in addition to alleviating weight loss and rejuvenating pulmonary health in experimental animals (94). These data show promising preclinical support for future studies investigating the clinical utility of TH5487 for treatment of IPF.

The approach of TH5487 targeting OGG1 to suppress fibrosis is distinct from currently employed nintedanib or pirfenidone. TH5487 inhibits intrahelical 8-oxoGua repair, raising issues concerned with genome fidelity and mutagenesis. Previous work reported that *Ogg1* KO mice develop normally, showing tolerable accumulation of 8-oxoGua in the non-proliferative tissues and a low frequency of malignancies (109–111). Through our LC-MS/MS DIA analysis (94), we identified that the back-up repair system is upregulated in TH5487-treated fibrotic lungs, among which the levels of Xeroderma pigmentosum complementation group C protein (XPC), X-ray repair cross-complementing protein1 (XRCC1), DNA polymerase delta subunit 2/3, DNA ligase 1, and G/T mismatch-specific thymine DNA glycosylase are increased. Presently, there is no data on any roles of these repair proteins in type II EMT or fibrosis, however, we note that the elevation of these repair proteins rather points to a back-up repair system that maintains genome integrity in the absence of functional OGG1. In addition to BER, the single strand break (SSB) repair pathway encompasses a spectrum of glycosylases that recognize 8-oxoGua mismatched with adenine, guanine, or thymine. For examples, human homolog of *E. coli* MutY, MYH, that flip out the normal adenine from the active site pocket instead of 8-oxoGua. Another example is *E. coli* Nei-like glycosylases (NEIL1 and NEIL2) (112–116). In the absence of OGG1, the activation of alternative repair pathways minimizes the mutagenic effects of elevated 8-oxoGua levels and maintains a low endogenous mutation frequency. These studies suggest that functional inactivation of OGG1 is not a major threat for cellular health. To this end, the non-toxic and well-tolerated TH5487, is a highly selective and specific OGG1 inhibitor offering clinical utility.

## 6 Concluding perspectives

8-oxoGua as an epigenetic mark read by OGG1 is captured as the underlying principle for the genomic response to oxidative stress. This epigenetic reprogramming induced by OGG1 modulates cell fate in response to environmental changes, including whether to undergo death or to survive through transition, and may ultimately result in complex phenotypes or disease conditions. The aforementioned correlations between the temporal and repurposed nuances in OGG1 behavior and transcription profiles in small airway epithelial cells likely influence the nature and magnitude of mucosal immune response. There is a transitioned cellular landscape maintained by OGG1 that is critical for amplifying the stress signal into mucosal responses. A better understanding of the molecular checkpoints that control these transitions might provide new insights for modulating immunity in pulmonary disorders. OGG1-targeted small molecules such as TH5487 have the potential to mitigate aberrant inflammatory responses and epithelium reepithelization through inhibition of NFκB activation and of the SMADs signaling pathway. Recent advances in single cell sequencing enabled us to identify cell phenotypes and to examine their proteomes and metabolomes in addition to surveying their epigenetic modifications. Mapping cell location in spatial transcriptomic techniques will undoubtedly enhance our understanding of cell molecular phenotype and function of transitional cell states driven by OGG1. Treating airways with small molecule OGG1 inhibitors will allow us to link molecular changes with disease phenotypes and could provide clinical relevance to decrease fibrotic processes in lung injury.

## Author contributions

LP and IB conceived, designed, and wrote the manuscript. SV, LT contributed to the writing and editing of the manuscript. AB, ZR, JB, AE, XB and ARB contributed to scientific advice, discussions about the generated network. All authors contributed to the article and approved the submitted version.
